# Plasma microRNA panels to diagnose pancreatic cancer: Results from a multicenter study

**DOI:** 10.18632/oncotarget.9491

**Published:** 2016-05-19

**Authors:** Zhe Cao, Chang Liu, Jianwei Xu, Lei You, Chunyou Wang, Wenhui Lou, Bei Sun, Yi Miao, Xubao Liu, Xiaowo Wang, Taiping Zhang, Yupei Zhao

**Affiliations:** ^1^ Department of General Surgery, Peking Union Medical College Hospital, Chinese Academy of Medical Sciences and Peking Union Medical College, Beijing, 100730, China; ^2^ MOE Key Laboratory of Bioinformatics, Bioinformatics Division and Center for Synthetic and Systems Biology, TNLIST/Department of Automation, Tsinghua University, Beijing, 100084, China; ^3^ Department of General Surgery, Qilu Hospital, Shandong University, Jinan, 250012, China; ^4^ Department of General Surgery, Pancreatic Disease Institute, Wuhan Union Hospital, Tongji Medical College, Huazhong University of Science and Technology, Wuhan, Hubei Province, 430022, China; ^5^ Department of Pancreatic Surgery, Zhong Shan Hospital, Fudan University, Shanghai, 200032, China; ^6^ Department of Hepatobiliary and Pancreatic Surgery, The First Affiliated Hospital, Harbin Medical University, Harbin, 150001, China; ^7^ Department of General Surgery, The First Affiliated Hospital, Nanjing Medical University, Nanjing, 210029, China; ^8^ Department of Hepatopancreatobiliary Surgery, West China Hospital, Sichuan University, Chengdu, Sichuan, 610041, China

**Keywords:** pancreatic cancer, microRNA panels, multicenter study, diagnosis

## Abstract

Biomarkers for the early diagnosis of pancreatic cancer (PC) are urgent needed. Plasma microRNAs (miRNAs) might be used as biomarkers for the diagnosis of cancer. We analyzed 361 plasma samples from 6 surgical centers in China and performed machine learning approach. We gain insight of the association between the aberrant plasma miRNA expression and pancreatic disease. 671 microRNAs were screened in the discovery phase and 33 microRNAs in the training phase and 13 microRNAs in the validation phase. After the discovery phase and training phase, 2 diagnostic panels were constructed comprising 3 microRNAs in panel I (miR-486-5p, miR-126-3p, miR-106b-3p) and 6 microRNAs in panel II (miR-486-5p, miR-126-3p, miR-106b-3p, miR-938, miR-26b-3p, miR-1285). Panel I and panel II had high accuracy for distinguishing pancreatic cancer from chronic pancreatitis (CP) with area under the curve (AUC) values of 0.891 (Standard Error (SE): 0.097) and 0.889 (SE: 0.097) respectively, in the validation phase. Additionally, we demonstrated that the diagnostic value of the panels in discriminating PC from CP were comparable to that of carbohydrate antigen 19–9 (CA 19–9) 0.775 (SE: 0.053) (*P* = 0.1 for both). This study identified 2 diagnostic panels based on microRNA expression in plasma with the potential to distinguish PC from CP. These patterns might be developed as biomarkers for pancreatic cancer.

## INTRODUCTION

Pancreatic cancer is a very lethal disease with the 5-year survival rate less than 5% [1]. Although surgical resection shows promise as an effective treatment for PC, Only 8–9% of pancreatic cancer patients can be diagnosed at an early stage [2]. Early diagnosis is the key strategy for improving the long-term outcome of pancreatic cancer.

Current methods for the diagnosis of PC can be divided into two main categories: imaging techniques and serological markers [3]. However, the diagnostic performance of these tests is unsatisfactory, particularly for the diagnosis of early-stage PC [4]. Carbohydrate antigen 19-9 (CA 19-9) has been used for many years as a serum marker for PC diagnosis [5]. However, it has been recognized that CA 19-9 has poor sensitivity in the detection of PC and that CA 19-9 levels often increase in the absence of PC (for example chronic pancreatitis or benign biliary obstruction) as well [6, 7]. Therefore, to improve the prognosis of PC, it is urgent to develop specific and noninvasive biomarkers for PC diagnosis, especially for early-stage tumors.

MicroRNAs are dysregulated in multiple tumors and are involved in the regulation of tumorigenesis and development [8–10], and several specific microRNA profiles related to pancreatic cancer tissue are described [11, 12]. miR-155 is abnormal during the Pancreatic Intraepithelial Neoplasia 2 (PanIN-2) stage, and miR-21 abnormalities occur during the PanIN-3 lesion stage [13, 14]. These studies indicate that miRNAs might be useful markers for the early diagnosis of pancreatic cancer [15].

In this study, we performed a machine learning approach to gain insight of the association between the aberrant plasma miRNA expression and pancreatic cancer, based on the three-phases, multicenter study. We analyzed the genome-wide expression of plasma miRNAs in pancreatic cancer, chronic pancreatitis patients and health control (HC) by a high-throughput technology and then developed 2 panels of plasma miRNAs using results from the training phase. Furthermore, the panels were comparable to CA19-9, as a marker for PC diagnosis. This highlights the diagnostic potential for noninvasive evaluation of pancreatic disorders.

## RESULTS

### Patient characteristics

The characteristics of the study participants are described previously [16]. There was no significant difference in the distribution of age and sex between the training and validation phases for the patients with pancreatic cancer and controls.

### MicroRNA screening and testing

In the discovery phase, multivariable analysis demonstrated that 15 microRNAs had the potential to separate patients with pancreatic cancer from healthy controls. Compared with patients with chronic pancreatitis, 19 miRNAs were significantly dysregulated in patients with pancreatic cancer [16].

### MicroRNA expression profile in the training phase

In the training phase, we validated the expression levels of 33 selected miRNAs (An additional four miRNAs: miR-126-3p, miR-19b-3p, miR-486-5p, and miR-942 were selected based on their potential diagnostic values for cancers [17–20]) with a *P* value of less than 0.05 (Student *t*-tests). Thirteen miRNAs were dysregulated including miR-106b-3p, miR-126-3p, miR-1271, miR-1285, miR-19b-3p, miR-26b-3p, miR-296-5p, miR-486-5p, miR-663B, miR-7-5p, miR-938, miR-942, and miR-181c-5p [16]. The multivariate *P* values for all of 13 microRNAs were < 0.05.

### Establishing the predictive MicroRNA panel

Based on the results from the training cohort, we noticed that three microRNAs combination could greatly improve the prediction of our classifier for diagnose, further increasing the microRNA numbers could slightly improve the accuracy with the maximum achieved by six microRNAs (Supplementary Figure S1). Two diagnostic panels were developed, Panel I was including miR-486-5p, miR-126-3p, miR-106b-3p, panel II was including miR-486-5p, miR-126-3p, miR-106b-3p, miR-938, miR-26b-3p, and miR-1285.

In the training phase to diagnose PC from CP, Panel I and panel II had high accuracy for distinguishing PC from CP with area under the curve (AUC) values of 0.906 (SE: 0.128) and 0.914 (SE: 0.126) respectively. The accuracy was 75.7% (SE, 0.176), sensitivity was 77.1% (SE, 0.232), specificity was 74.3% (SE, 0.284) for panel I. And the accuracy was 82.3% (SE, 0.147), sensitivity was 83.9% (SE, 0.203), specificity was 80.8% (SE, 0.237) for panel II (Table 1). The box plots of support vector machine (SVM) decision value of panel I and II using the plasma samples were shown in Figure 2.

**Table 1:** Performance of panel I and II and CA 19-9 in the differential diagnosis of pancreatic cancer from chronic pancreatitis (CP) and other pancreatic neoplasms (OPN) in training phase and validation phase

Test and type of patients by phase	ID	Accuracy	SE	Sensitivity	SE	Specificity	SE	AUC	SE
**Training phase**									
Pancreatic cancer vs. Chronic pancreatitis	Panel I	0.757	0.176	0.771	0.232	0.743	0.284	0.906	0.128
	Panel II	**0.823**	0.147	**0.839**	0.203	**0.808**	0.237	**0.914**	0.126
**Validation phase**									
Pancreatic cancer vs. Chronic pancreatitis	Panel I	**0.836**	0.109	**0.827**	0.165	**0.844**	0.162	**0.891**	0.097
	Panel II	0.818	0.115	0.823	0.17	0.814	0.176	0.889	0.097
	CA 19-9	0.794	0.049	0.759	0.128	0.829	0.075	0.775	0.053
Pancreatic cancer vs. Other pancreatic neoplasms	Panel I	0.539	0.162	0.568	0.237	0.51	0.268	0.677	0.142
	Panel II	0.649	0.148	0.648	0.214	0.649	0.23	0.737	0.147
	CA 19-9	**0.853**	0.028	**0.749**	0.073	**0.957**	0.055	**0.86**	0.031
Chronic pancreatitis vs. Other pancreatic neoplasms	Panel I	0.652	0.141	0.636	0.225	0.668	0.216	0.752	0.251
	Panel II	**0.715**	0.13	**0.65**	0.21	0.779	0.192	**0.79**	0.142
	CA 19-9	0.646	0.033	0.432	0.153	**0.86**	0.147	0.626	0.091

The accuracy, sensitivity, specificity and AUC were all estimated by bootstrapping method.

### Validating the MicroRNA panel

The panels estimated from the training phase were used to predict the probability of being diagnosed with pancreatic cancer for the independent validation phase (298 plasma samples).

Panel I and panel II showed diagnostic value in discriminating PC from CP with AUC values of 0.891 (SE: 0.097) and 0.889 (SE: 0.097) respectively, and accuracy value of 83.6% (SE: 0.109), and 81.8% (SE: 0.116, respectively (Table 1).

Panel I and panel II displayed diagnostic value in discriminating PC from patients with other pancreatic neoplasms (OPN), with AUC values of 0.677 (SE: 0.142) and 0.737 (SE: 0.147) respectively, accuracy of 53.9% (SE: 0.162), and 64.9% (SE: 0.148), respectively (Table 1).

Panel I and panel II displayed diagnostic value in discriminating CP from OPN with AUC values of 0.752 (SE: 0.251) and 0.790 (SE: 0.142) respectively, accuracy of 65.2% (SE: 0.141), and 71.5% (SE: 0.130) (Table 1).

### Comparison of the diagnostic values of the microRNA panels with CA 19-9

**Figure 1:**
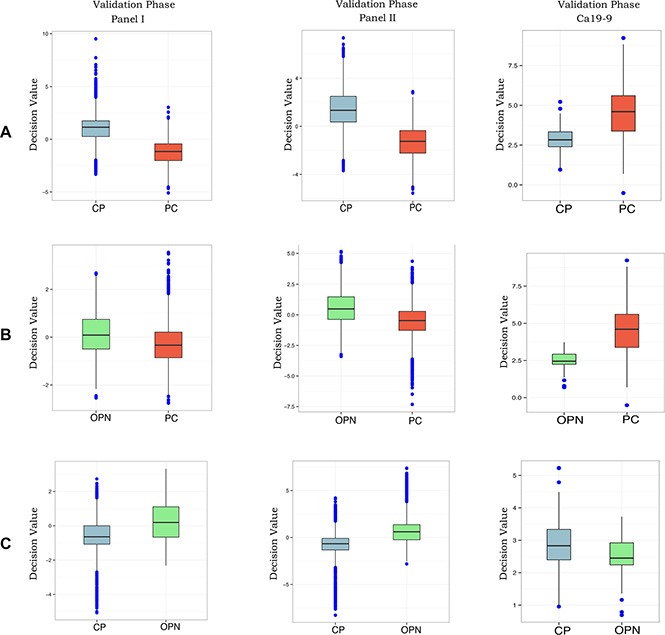
Box plots of panel I and II and CA 19-9 using the plasma samples from the validation phase The decision value of Panel I and Panel II was calculated by SVM with the bootstrap method (ten was left for testing and the other was for the model) and the decision value of Ca19-9 is just the expression value.

We also examined CA 19-9 levels (Table 1) and compared the diagnostic value of the miRNA panels with the CA 19-9. We demonstrated that the AUC value of panel I and panel II were comparable to CA 19-9 when discriminating patients with PC from CP (*P* = 0.1 and *P* = 0.1, respectively). The AUC value of panel II was comparable to CA 19-9 when discriminating CP from OPN (*P* = 0.1, Table 2). The box plots of SVM decision value of panel I and II (also Ca19-9 expression value) using the plasma samples were shown in Figure 1.

**Table 2:** Comparison of the diagnostic power of the microRNA panels with CA 19-9 in the validation phase

Group	Panel ID	AUC1 (panels)	SE1^*^ (panels)	AUC2 (CA 19-9)	SE2^*^ (CA 19-9)	*Z*-value	*P*-value
Pancreatic cancer vs.Chronic pancreatitis	Panel I	0.891	0.097	0.775	0.053	1.05	0.1
	Panel II	0.889	0.097	0.775	0.053	1.04	0.1
Pancreatic cancer vs. Other pancreatic neoplasms	Panel I	0.677	0.142	0.860	0.031	−1.27	0.1
	Panel II	0.737	0.147	0.860	0.031	−0.82	0.2
Chronic pancreatitis vs. Other pancreatic neoplasms	Panel I	0.752	0.251	0.626	0.091	0.47	0.3
	Panel II	0.790	0.091	0.626	0.091	1.27	0.1

* The SE (standard error) of two panels were estimated by the bootstrapping method

**Figure 2:**
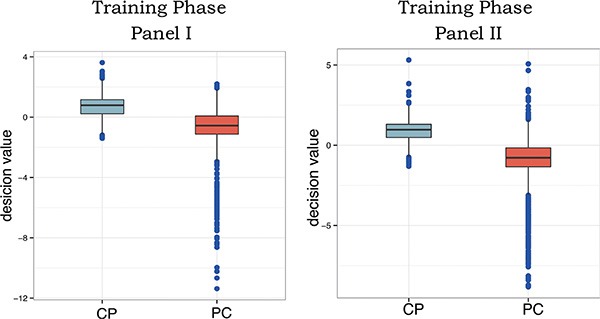
Box plots of SVM decision of panel I and II using the plasma samples from the training phase The decision value was calculated by SVM with the bootstrap method (six was left for testing and the other was for the model) and the bigger the difference of decision value, the easier to diagnose between the two diseases.

## DISCUSSION

Sensitive and specific biomarkers to identify patients with pancreatic cancer at an early stage are needed [21–23]. This study describes 2 novel panels of miRNAs for diagnosing pancreatic cancer using the combination of 3 or 6 miRNAs in plasma.

The plasma microRNA candidates were selected in the discovery phase using microarray, which comprised a total of 671 miRNAs. Two panels were then developed using results from the training phase and predicted the probability of being diagnosed with pancreatic cancer in the independent validation phase.

Panel I was comparable to panel II when discriminating pancreatic cancer from chronic pancreatitis. However, panel II was better than panel I when discriminating pancreatic cancer from other pancreatic neoplasms. These results indicated that the diagnostic value of plasma miRNAs was sensitive with different pancreatic disease.

In our previous study [16], we found miR-486-5p was able to discriminate PC patients from CP patients. Here, in this study, the plasma miRNA panels discovered in this study (panel I:AUC 0.891, panel II:AUC 0.889) showed better performance than CA19-9 (AUC 77.5%) and miR-486-5p (AUC 73.8%) (Supplementary Table S1). Though limited by current sample size, the difference is not statistical significant. (*p*-value = 0.14 and 0.08, respectively). Studies in larger sample size are required to further clarify whether these panels could work as a better diagnostic tool for pancreatic cancer than CA 19-9. The miRNA panels may serve as a novel noninvasive approach for diagnosis of pancreatic cancer. This approach might be applicable for patients with biliary obstruction and Lewis-genotype negative particularly. Studies in more sample size are required to clarify the relevance and significance of the panels as a possible diagnostic tool for pancreatic cancer.

Liu, et al. [24] reported a microRNA classifier in serum for pancreatic cancer with an accuracy of 83.6%, which was higher than CA 19-9 (56.4%), and a combination of 2 microRNAs in plasma with serum CA 19-9 (AUC of 0.98), but this panel has not been validated [25]. Schultz, et al. [26] found two diagnostic panels based on miRNA expression in whole blood that showed potential for distinguishing pancreatic cancer from chronic pancreatitis in a multicenter study. However, other pancreatic neoplasms (such as pancreatic neuroendocrine neoplasm (PNEN), serous or mucinous cystadenomas, solid pseudopapillary tumors, intraductal papillary mucinous neoplasm (IPMN).) were not included in their study. Li, et al. [27] demonstrated that serum miR-1290 showed potential when distinguishing pancreatic cancer from chronic pancreatitis, PNEN, and IPMN. However, all of the samples were collected from a single center. The strengths of the current study are the relatively large number of pancreatic patients and controls over three phases and 6 surgical centers. The large number of microRNAs analyzed in the discovery phase for selection of microRNA candidates, and the validation of the panels in 2 other populations using another assay platform. The statistical precision is thus evaluated in terms of magnitude of confidence intervals of effect estimates or standard error and in terms of predictive ability of the proposed panels. To the best of our knowledge, the current study is the first multicenter trial performing machine learning strategy to describe differences in plasma miRNA expression between patients with pancreatic cancer and chronic pancreatitis and other pancreatic neoplasms.

Several miRNAs in the panels, which observed to be dysregulated in the current study are associated with tumor or stem cell biology. MiR-486-5p is dysregulated in many types of cancer and is involved in NF-kB signaling and in CD40 pathways [28]. MiR-486-5p is activate in pancreatic cancer tissues compared with normal tissues [17], and associated with invasion and metastasis [28]. The miR-126-3p has been found to suppress cell invasion, metastasis [29, 30], and also dysregulation in plasma from patients with pancreatic and prostate cancer [26, 31]. Overexpression of miR-938 has been associated with chemoresistant glioblastomas [32]. Butz, et al. [33] predicted SMAD3 may be the target of miR-938 in pituitary adenomas, which can decrease TGF-β pathway activation, leading more activated pathway like Ras-MAPK, c-Jun and PI3K-Akt, which are proved the core pathways in pancreatic cancer [34–38].

One limitation of our study is that as the number of healthy control was limited, no panel could be found to have a better diagnosed power between patients with pancreatic cancer and healthy controls. Our study should therefore be seen as an exploratory study. Another limitation of the current study is that the diagnostic value of combining the miRNAs with CA 19-9 was not evaluated because not all the plasma samples had tested CA 19-9.

Pancreatic cancer is a very lethal disease, and today most patients are diagnosed too late for surgery to be performed [39, 40]. Because patients with early-stage pancreatic cancer generally can undergo complete resection of tumors, the current study would refer more patients with characteristic or uncharacteristic symptoms to CT, magnetic resonance, or ultrasound imaging. The test of plasma miRNAs could thereby diagnose more patients with pancreatic cancer, some of them at an early stage, and thus have a potential to increase the number of early pancreatic cancer patients that can be operated on and possibly cured of pancreatic cancer.

## MATERIALS AND METHODS

### Patient samples

Patients with pancreatic disease who were treated in surgical departments at 6 hospitals in China were included in our previous study [16]. PC was diagnosed on the basis of cytological or histological examinations. CP was diagnosed based on clinical diagnostic criteria [41] or histological examinations. OPN were diagnosed based on histological examinations. OPN included PNEN, serous or mucinous cystadenomas, solid pseudopapillary tumors, IPMN or epithelial cysts.

The patients included in this study were all consecutive patients who met inclusion criteria and agreed to participate. Blood samples were taken before treatment. This study was approved by the Institutional Review Board of Peking Union Medical College Hospital. Written informed consent was obtained from all of the patients.

**Figure 3:**
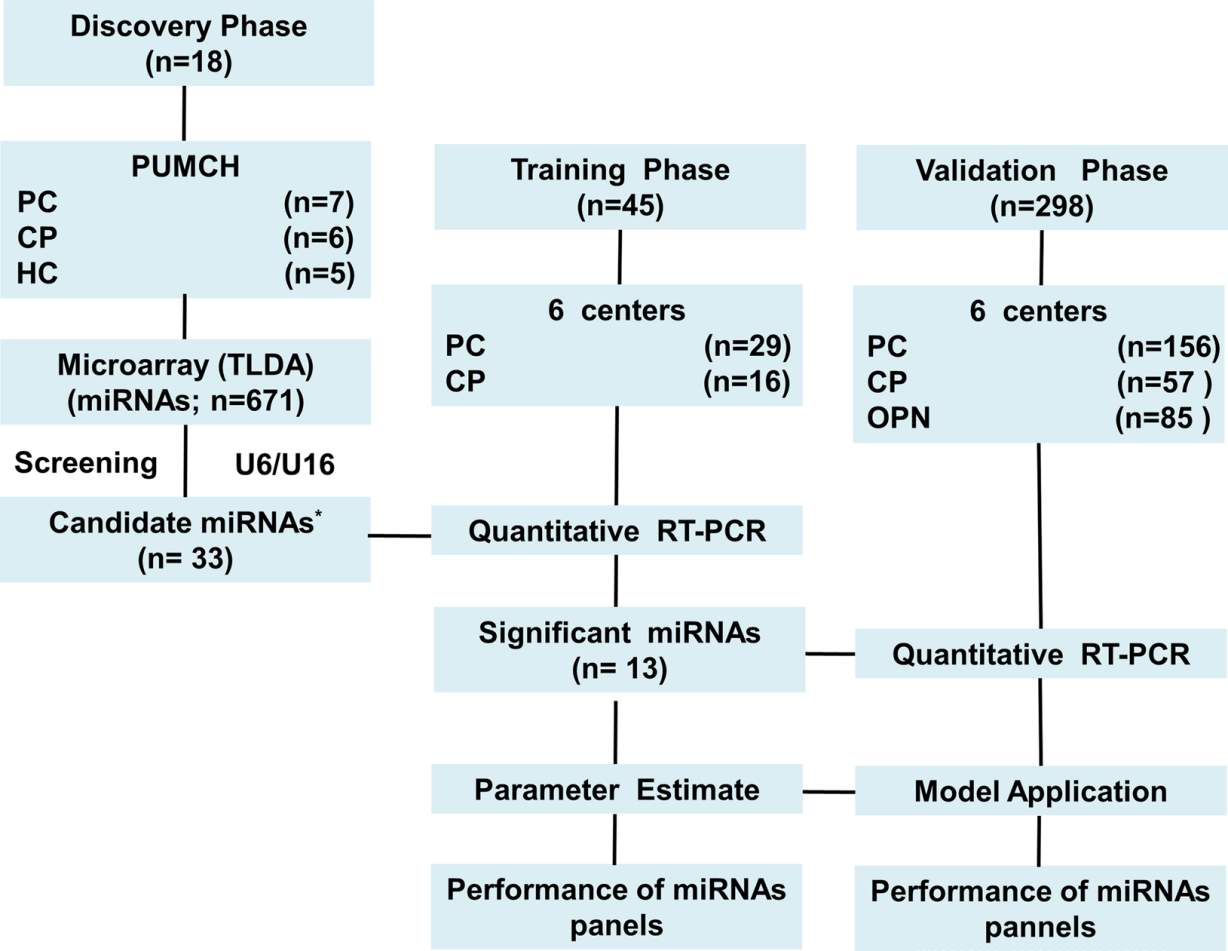
Study design PUMCH, peking union medical college hospital; PC, pancreatic cancer; CP chronic pancreatitis; HC, healthy controls; OPN, other pancreatic neoplasms; RT-PCR, reverse transcriptase polymerase chain reaction. *An additional four miRNAs: miR-126-3p, miR-19b-3p, miR-486-5p, and miR-942 were selected based on their potential diagnostic values for cancers.

Details of the design of the study appear in Figure 3. Pretreatment blood samples from 7 patients with pancreatic cancer, 6 patients with chronic pancreatitis and 5 healthy controls were allocated to the discovery phase from Peking Union Medical College Hospital (PUMCH). MiRNA microarray were used to detect the miRNA level in these samples. Then, pretreatment blood samples from 185 patients with pancreatic cancer, 73 patients with chronic pancreatitis and 85 patients with other pancreatic neoplasms, were allocated in chronological order to the training phase and validation phase from all 6 participating centers: 1) Peking Union Medical College Hospital, Beijing; 2) Union Hospital, Tongji Medical College, Huazhong University of Science and Technology, Wuhan; 3) Zhongshan Hospital, Fudan University, Shanghai; 4) First Affiliated Hospital of Harbin Medical University, Harbin; 5) First Affiliated Hospital with Nanjing Medical University, Nanjing; and 6) Sichuan Provincial Pancreatitis Centre, West China Hospital, Sichuan University, Chengdu. We used the discovery phase for screening (ie, reducing the number of candidate microRNAs for further investigation). Potential microRNAs were measured in the training phase and used for the derivation of diagnostic panels. In addition, the predictive performance was investigated in the validation phase.

Details for the miRNA microarray analysis (Taqman low density arrays, TLDA, Applied Biosystems, Life Technologies, Shanghai, China), miRNA isolation and quantitative real-time polymerase chain reaction (qRT-PCR) analysis are described previously [16].

Serum samples from 271 subjects including 144 patients with pancreatic cancer, 31 with chronic pancreatitis, and 58 patients with other pancreatic neoplasms in validation phase were collected for detecting CA19-9 levels, using CA 19-9 enzyme-linked immuno sorbent assay (ELISA) kit (CSB-E04773h, CUSABIO, Wuhan, China) according to the protocol supplied by the manufacturer.

### Statistical analysis

qRT-PCR measured microRNA expression value was first normalized by U6. The two sample *t*-test was used firstly to select the significant microRNA between PC, HC and CP in each phase. And all the expression value was normalized by z-score transformation. Then, a SVM classifier based sequential forward feature selection approach was used to find the microRNAs panels for diagnosing PC and CP in the training phase (Supplementary Figure S1). We used linear kernel SVM implemented in the R package ‘e1071’ to train the model. The decision value was calculated by $\overset{\to T}{w}\vec{x}+w_{0}$, where $\vec{w}$ is the weight vector, *w*_0_ is a constant variable and $\vec{x}$ is the vector of expression level of microRNAs. And we infer that patient to be positive or negative by the decision value.

We perform the validation step by the bootstrap method to assess the generalization ability of our panel in the validation phase. (Supplementary Figure S2) We randomly leave ten samples to test the performance and use the other to train the parameters. We repeated this process for 1000 times to estimate the standard error. As the patient number of each disease is unbalanced, we sampled both set to get the same number of positive and negative patients. And the ROC curve was drawn by the decision value of the SVM model and evaluate the diagnostic effectiveness of the panel by AUC.

Area under curve (AUC) of panels (AUC1) and CA 19-9 (AUC2) were compared using the R package ‘pROC’ with the Delong option [42]. And the test was given below as *Z* value=|AUC1-AUC2|/sqrt(SE1^2+SE2^2), *P* value=1-NormDist(Z).

## CONCLUSIONS

This study identified 2 diagnostic miRNA panels in plasma that had the ability to distinguish, to a certain degree, patients with pancreatic cancer from chronic pancreatitis and other pancreatic neoplasms. Although we validated the panels, our findings are preliminary. Further research is necessary to understand whether these miRNAs have clinical implications as a screening test for early detection of pancreatic cancer and how much this information adds to serum CA 19-9.

### List of genes

miR-486-5p, miR-938, miR-126-3p, miR-26b-3p, miR-1285 and miR-106b-3p.

## SUPPLEMENTARY MATERIALS FIGURES AND TABLES



## Abbreviations

miRNA: microRNA
PC: pancreatic cancer
CP: chronic pancreatitis
HC: healthy control
OPN: Other pancreatic neoplasms
CA 19-9: carbohydrate antigen 19-9
qPCR: quantitative real-time PCR
AUC: area under the curve
ROC: Receiver-operator characteristic
SE: standard Error
SVM: support vector machine

## References

[1] Siegel RL, Miller KD, Jemal A (2015). Cancer statistics 2015. CA Cancer J Clin.

[2] Costello E, Neoptolemos JP (2011). Pancreatic cancer in 2010: new insights for early intervention and detection. Nature reviews Gastroenterology & hepatology.

[3] Paulson AS, Tran Cao HS, Tempero MA, Lowy AM (2013). Therapeutic advances in pancreatic cancer. Gastroenterology.

[4] Sohal DP, Walsh RM, Ramanathan RK, Khorana AA (2014). Pancreatic adenocarcinoma: treating a systemic disease with systemic therapy. Journal of the National Cancer Institute.

[5] Atkins CD (2009). CA 19–9 and Lewis antigens in pancreatic cancer. Journal of clinical oncology.

[6] von Rosen A, Linder S, Harmenberg U, Pegert S (1993). Serum levels of CA 19-9 and CA 50 in relation to Lewis blood cell status in patients with malignant and benign pancreatic disease. Pancreas.

[7] Winter JM, Yeo CJ, Brody JR (2013). Diagnostic prognostic and predictive biomarkers in pancreatic cancer. Journal of surgical oncology.

[8] Iorio MV, Croce CM (2012). MicroRNA dysregulation in cancer: diagnostics monitoring and therapeutics A comprehensive review. EMBO molecular medicine.

[9] Chitkara D, Mittal A, Mahato RI (2015). miRNAs in pancreatic cancer: therapeutic potential delivery challenges and strategies. Advanced drug delivery reviews.

[10] Wang P, Zhang L, Chen Z, Meng Z (2013). MicroRNA targets autophagy in pancreatic cancer cells during cancer therapy. Autophagy.

[11] Schultz NA, Werner J, Willenbrock H, Roslind A, Giese N, Horn T, Wojdemann M, Johansen JS (2012). MicroRNA expression profiles associated with pancreatic adenocarcinoma and ampullary adenocarcinoma. Modern pathology.

[12] Szafranska AE, Doleshal M, Edmunds HS, Gordon S, Luttges J, Munding JB, Barth RJ, Gutmann EJ, Suriawinata AA, Marc Pipas J, Tannapfel A, Korc M, Hahn SA (2008). Analysis of microRNAs in pancreatic fine-needle aspirates can classify benign and malignant tissues. Clinical chemistry.

[13] Ryu JK, Hong SM, Karikari CA, Hruban RH, Goggins MG, Maitra A (2010). Aberrant MicroRNA-155 expression is an early event in the multistep progression of pancreatic adenocarcinoma. Pancreatology.

[14] du Rieu MC, Torrisani J, Selves J, Al Saati T, Souque A, Dufresne M, Tsongalis GJ, Suriawinata AA, Carrere N, Buscail L, Cordelier P (2010). MicroRNA-21 is induced early in pancreatic ductal adenocarcinoma precursor lesions. Clinical chemistry.

[15] Schwarzenbach H, Nishida N, Calin GA, Pantel K (2014). Clinical relevance of circulating cell-free microRNAs in cancer. Nature reviews Clinical oncology.

[16] Xu J, Cao Z, Liu W, You L, Zhou L, Wang C, Lou W, Sun B, Miao Y, Liu X, Zhang T, Zhao Y (2016). Plasma miRNAs Effectively Distinguish Patients With Pancreatic Cancer From Controls: A Multicenter Study. Annals of surgery.

[17] Ali S, Saleh H, Sethi S, Sarkar FH, Philip PA (2012). MicroRNA profiling of diagnostic needle aspirates from patients with pancreatic cancer. British journal of cancer.

[18] Patnaik SK, Yendamuri S, Kannisto E, Kucharczuk JC, Singhal S, Vachani A (2012). MicroRNA expression profiles of whole blood in lung adenocarcinoma. PloS one.

[19] Fassina A, Marino F, Siri M, Zambello R, Ventura L, Fassan M, Simonato F, Cappellesso R (2012). The miR-17-92 microRNA cluster: a novel diagnostic tool in large B-cell malignancies. Laboratory investigation.

[20] Vosa U, Vooder T, Kolde R, Vilo J, Metspalu A, Annilo T (2013). Meta-analysis of microRNA expression in lung cancer. International journal of cancer.

[21] Ko AH (2015). Progress in the treatment of metastatic pancreatic cancer and the search for next opportunities. Journal of clinical oncology.

[22] Costello E, Greenhalf W, Neoptolemos JP (2012). New biomarkers and targets in pancreatic cancer and their application to treatment. Nature reviews Gastroenterology & hepatology.

[23] Rachagani S, Macha MA, Heimann N, Seshacharyulu P, Haridas D, Chugh S, Batra SK (2015). Clinical implications of miRNAs in the pathogenesis diagnosis and therapy of pancreatic cancer. Advanced drug delivery reviews.

[24] Liu R, Chen X, Du Y, Yao W, Shen L, Wang C, Hu Z, Zhuang R, Ning G, Zhang C, Yuan Y, Li Z, Zen K (2012). Serum microRNA expression profile as a biomarker in the diagnosis and prognosis of pancreatic cancer. Clinical chemistry.

[25] Liu J, Gao J, Du Y, Li Z, Ren Y, Gu J, Wang X, Gong Y, Wang W, Kong X (2012). Combination of plasma microRNAs with serum CA19-9 for early detection of pancreatic cancer. International journal of cancer.

[26] Schultz NA, Dehlendorff C, Jensen BV, Bjerregaard JK, Nielsen KR, Bojesen SE, Calatayud D, Nielsen SE, Yilmaz M, Hollander NH, Andersen KK, Johansen JS (2014). MicroRNA biomarkers in whole blood for detection of pancreatic cancer. Jama.

[27] Li A, Yu J, Kim H, Wolfgang CL, Canto MI, Hruban RH, Goggins M (2013). MicroRNA array analysis finds elevated serum miR-1290 accurately distinguishes patients with low-stage pancreatic cancer from healthy and disease controls. Clinical cancer research.

[28] Mees ST, Mardin WA, Sielker S, Willscher E, Senninger N, Schleicher C, Colombo-Benkmann M, Haier J (2009). Involvement of CD40 targeting miR-224 and miR-486 on the progression of pancreatic ductal adenocarcinomas. Annals of surgical oncology.

[29] Tai HC, Chang AC, Yu HJ, Huang CY, Tsai YC, Lai YW, Sun HL, Tang CH, Wang SW (2014). Osteoblast-derived WNT-induced secreted protein 1 increases VCAM-1 expression and enhances prostate cancer metastasis by down-regulating miR-126. Oncotarget.

[30] Wang CZ, Yuan P, Li Y (2015). MiR-126 regulated breast cancer cell invasion by targeting ADAM9. International journal of clinical and experimental pathology.

[31] Watahiki A, Macfarlane RJ, Gleave ME, Crea F, Wang Y, Helgason CD, Chi KN (2013). Plasma miRNAs as biomarkers to identify patients with castration-resistant metastatic prostate cancer. International journal of molecular sciences.

[32] Yan W, Liu Y, Yang P, Wang Z, You Y, Jiang T (2015). MicroRNA profiling of Chinese primary glioblastoma reveals a temozolomide-chemoresistant subtype. Oncotarget.

[33] Butz H, Liko I, Czirjak S, Igaz P, Korbonits M, Racz K, Patocs A (2011). MicroRNA profile indicates downregulation of the TGFbeta pathway in sporadic non-functioning pituitary adenomas. Pituitary.

[34] Williams TM, Flecha AR, Keller P, Ram A, Karnak D, Galban S, Galban CJ, Ross BD, Lawrence TS, Rehemtulla A, Sebolt-Leopold J (2012). Cotargeting MAPK and PI3K signaling with concurrent radiotherapy as a strategy for the treatment of pancreatic cancer. Molecular cancer therapeutics.

[35] Baumgart S, Chen NM, Siveke JT, Konig A, Zhang JS, Singh SK, Wolf E, Bartkuhn M, Esposito I, Hessmann E, Reinecke J, Nikorowitsch J, Brunner M (2014). Inflammation-induced NFATc1-STAT3 transcription complex promotes pancreatic cancer initiation by KrasG12D. Cancer discovery.

[36] Briest F, Grabowski P (2014). PI3K-AKT-mTOR-signaling and beyond: the complex network in gastroenteropancreatic neuroendocrine neoplasms. Theranostics.

[37] Sharma N, Nanta R, Sharma J, Gunewardena S, Singh KP, Shankar S, Srivastava RK (2015). PI3K/AKT/mTOR and sonic hedgehog pathways cooperate together to inhibit human pancreatic cancer stem cell characteristics and tumor growth. Oncotarget.

[38] Soares HP, Ming M, Mellon M, Young SH, Han L, Sinnet-Smith J, Rozengurt E (2015). Dual PI3K/mTOR Inhibitors Induce Rapid Overactivation of the MEK/ERK Pathway in Human Pancreatic Cancer Cells through Suppression of mTORC2. Molecular cancer therapeutics.

[39] Heestand GM, Murphy JD, Lowy AM (2015). Approach to patients with pancreatic cancer without detectable metastases. Journal of clinical oncology.

[40] Wolfgang CL, Herman JM, Laheru DA, Klein AP, Erdek MA, Fishman EK, Hruban RH (2013). Recent progress in pancreatic cancer. CA Cancer J Clin.

[41] Conwell DL, Lee LS, Yadav D, Longnecker DS, Miller FH, Mortele KJ, Levy MJ, Kwon R, Lieb JG, Stevens T, Toskes PP, Gardner TB, Gelrud A (2014). American Pancreatic Association Practice Guidelines in Chronic Pancreatitis: evidence-based report on diagnostic guidelines. Pancreas.

[42] Robin X, Turck N, Hainard A, Tiberti N, Lisacek F, Sanchez JC, Muller M (2011). pROC: an open-source package for R and S+ to analyze and compare ROC curves. BMC bioinformatics.

