# Supplementary material for: Plasma microRNA panels to diagnose pancreatic cancer: Results from a multicenter study
Source: Oncotarget. 2016 May 19;7(27):41575–83. doi: 10.18632/oncotarget.9491 (PMC5173079; doi:10.18632/oncotarget.9491)
Supplement: Supplementary file 1 [file oncotarget-07-41575-s001.pdf]

Plasma microRNA panels to diagnose pancreatic cancer: Results from a multicenter study

Supplementary Materials

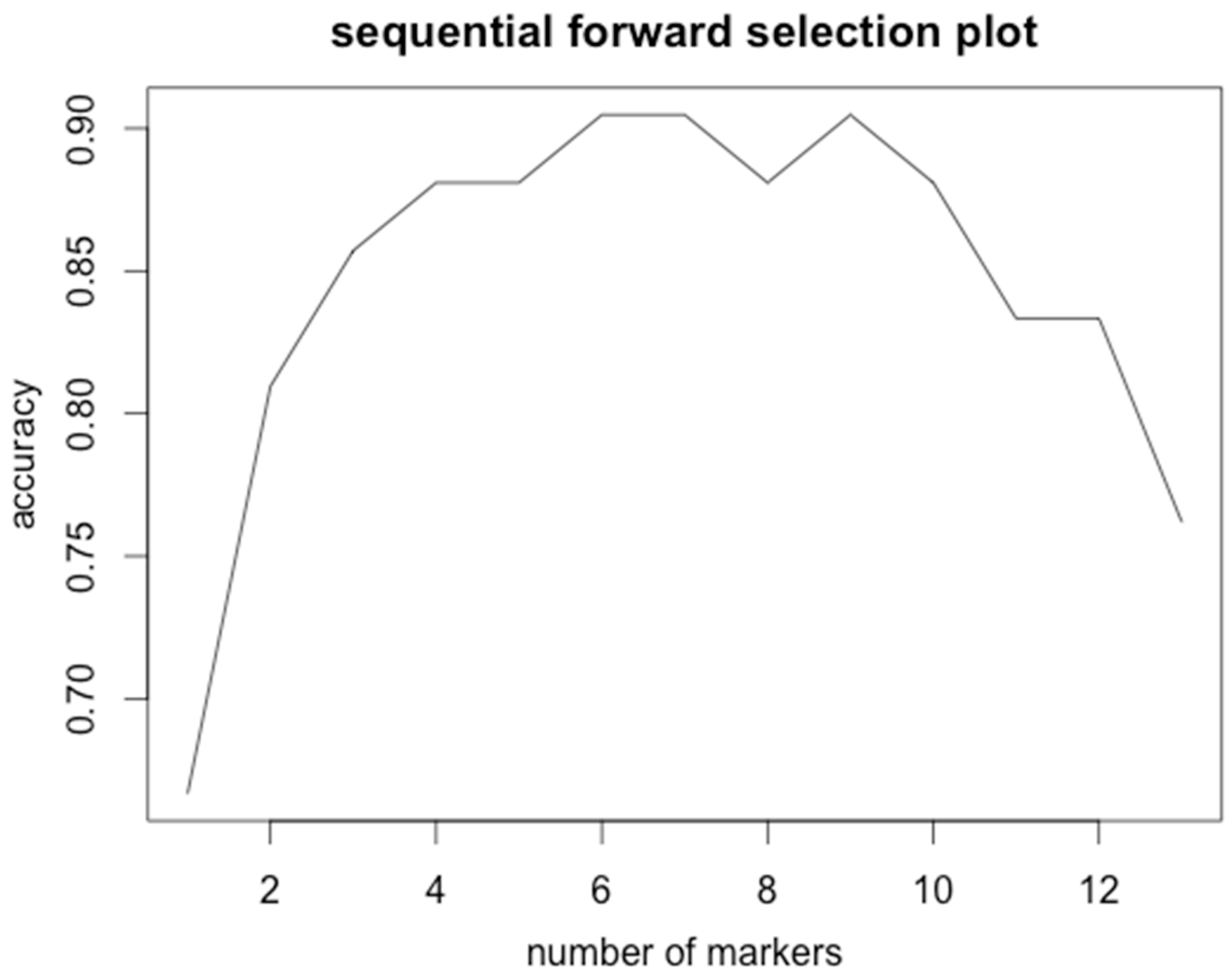

Supplementary Figure S1: The diagnostic value of multiple biomarkers in training phase. SVM based Sequential forward feature selection were used to select the best multiple biomarkers for diagnosing. Shown in the figure, three microRNAs combination could greatly improve the prediction (the elbow point of the curve) of our classifier for diagnose, further increasing the microRNA numbers could slightly improve the accuracy with the maximum achieved by six microRNAs. The diagnose ability was evaluated by leave-one-out approach.

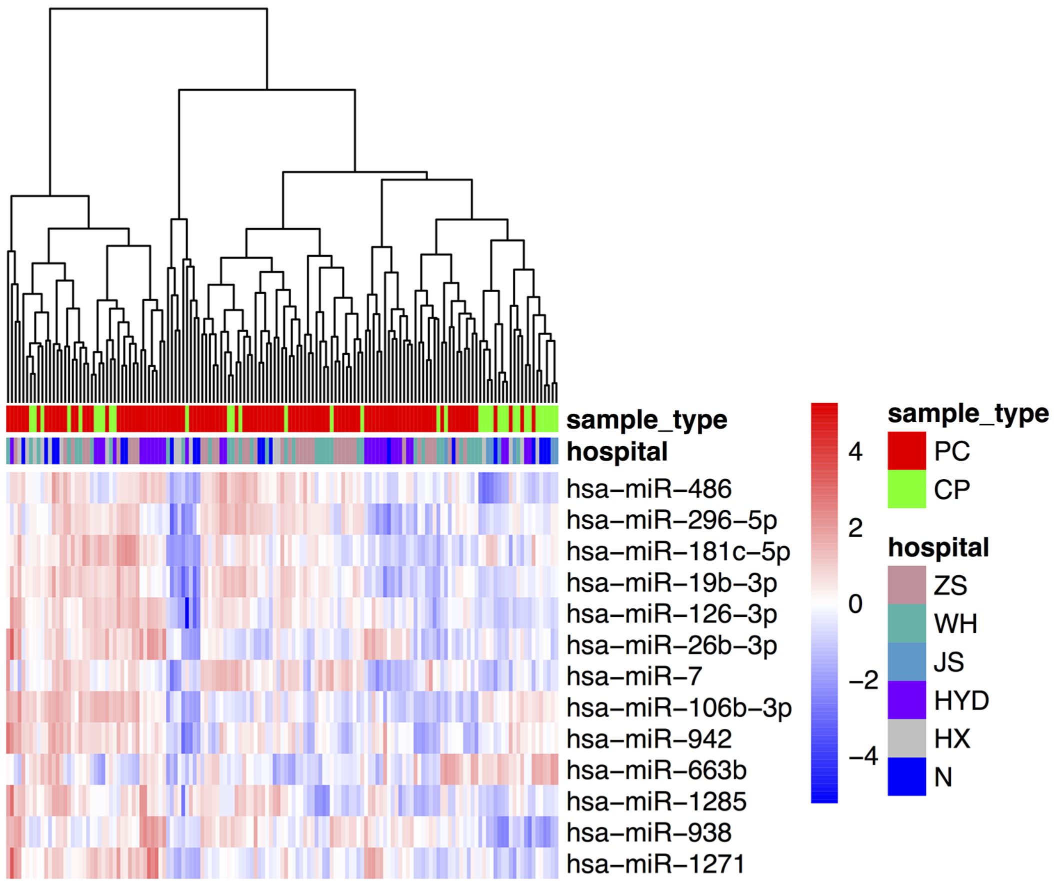

Supplementary Figure S2: Hieratical clustering of samples in validation phase. MicroRNA expression levels were normalized to U6 RNA level to eliminate batch effect. The hospital name is abbreviated as following: ZS (Zhongshan Hospital), WH (Wuhan Union Hospital), JS (First Affiliated Hospital with Nanjing Medical University), HYD (First Affiliated Hospital of Harbin Medical University), HX (West China Hospital), N (Peking Union Medical College Hospital).

Supplementary Table S1: Comparison of the diagnostic power of the microRNA panels with miR- 486- 5p in the validation phase

Group	Panel ID	AUC1 (panels)	SE1* (panels)	AUC2 (miR-486-5p)	SE2* (miR-486-5p)	*Z*-value	*P*-value
**Pancreatic cancer vs. Chronic pancreatitis**	Panel I	0.891	0.097	0.738	0.051	1.40	0.08
	Panel II	0.889	0.097	0.738	0.051	1.38	0.08
**Pancreatic cancer vs. Other pancreatic neoplasms**	Panel I	0.677	0.142	0.532	0.064	0.26	0.40
	Panel II	0.737	0.147	0.532	0.064	0.37	0.36
**Chronic pancreatitis vs. Other pancreatic neoplasms**	Panel I	0.752	0.251	0.683	0.067	0.10	0.46
	Panel II	0.790	0.091	0.683	0.067	0.16	0.44

*The SE (standard error) of two panels were estimated by the bootstrapping method.
